# Massive Bleeding After Surgical Repair in Acute Type A Aortic Dissection Patients: Risk Factors, Outcomes, and the Predicting Model

**DOI:** 10.3389/fcvm.2022.892696

**Published:** 2022-07-08

**Authors:** Chen-Han Zhang, Yi-Peng Ge, Yong-Liang Zhong, Hai-Ou Hu, Zhi-Yu Qiao, Cheng-Nan Li, Jun-Ming Zhu

**Affiliations:** Department of Cardiovascular Surgery, Beijing Aortic Disease Center, Beijing Anzhen Hospital, Beijing Institute of Heart Lung and Blood Vessel Diseases, Capital Medical University, Beijing, China

**Keywords:** aortic (rupture) dissection, bleeding, perioperative management, surgery strategy, clinical research

## Abstract

**Background:**

Massive bleeding throughout aortic repair in acute type A aortic dissection (ATAAD) patients is a common but severe condition that can cause multiple serious clinical problems. Here, we report our findings regarding risk factors, short-term outcomes, and predicting model for massive bleeding in ATAAD patients who underwent emergent aortic repair.

**Methods:**

A universal definition of perioperative bleeding (UDPB) class 3 and 4 were used to define massive bleeding and comprehensively evaluate patients. A total of 402 consecutive patients were enrolled in this retrospective study during 2019. Surgical strategies used to perform aortic arch procedures included total arch and hemiarch replacements. In each criterion, patients with massive bleeding were compared with remaining patients. Multivariable regression analyses were used to identify independent risk factors for massive bleeding. Logistic regression was used to build the model, and the model was evaluated with its discrimination and calibration.

**Results:**

Independent risk factors for massive bleeding included male sex (OR = 6.493, *P* < 0.001), elder patients (OR = 1.029, *P* = 0.05), low body mass index (BMI) (OR = 0.879, *P* = 0.003), emergent surgery (OR = 3.112, *P* = 0.016), prolonged cardiopulmonary bypass time (OR = 1.012, *P* = 0.002), lower hemoglobin levels (OR = 0.976, *P* = 0.002), increased D-dimer levels (OR = 1.000, *P* = 0.037), increased fibrin degradation products (OR = 1.019, *P* = 0.008), hemiarch replacement (OR = 5.045, *P* = 0.037), total arch replacement (OR = 14.405, *P* = 0.004). The early-stage mortality was higher in massive bleeding group (15.9 vs. 3.9%, *P* = 0.001). The predicting model showed a well discrimination (AUC = 0.817) and calibration (χ^2^ = 5.281, *P* = 0.727 > 0.05).

**Conclusion:**

Massive bleeding in ATAAD patients who underwent emergent aortic repair is highly associated with gender, emergent surgery, increased D-dimer levels, longer CPB time, anemia, and use of a complex surgical strategy. Since massive bleeding may lead to worse outcomes, surgeons should choose suitable surgical strategies in patients who are at a high risk of massive bleeding.

## Introduction

Acute type A aortic dissection (ATAAD) is one of the most complex and life-threatening disorders and can lead to catastrophic results, such as aortic rupture or even death (1). Medically managed ATAAD patients are at risk of poor outcomes, and mortality rates in these patients reach 20, 30, and 50% in the first 24 h after acute presentation, after 48 h, and after 1 month, respectively (2). Therefore, emergent aortic repair is the best treatment option for ATAAD and is potentially life-saving. Despite improvements in patient diagnosis and surgical techniques, aortic repair is still associated with high morbidity and mortality rates compared with other cardiac procedures (3, 4). Among all possible complications of aortic surgery, postoperative bleeding is one of the most prevalent and intractable. In particular, when the hemorrhage is massive, failed or delayed treatment may cause irreversible organ dysfunction, such as renal failure, cardiovascular events including myocardial injury, or even death (5). Therefore, a full understanding of ATAAD risk factors can significantly benefit clinical practice. Many factors may be related to postoperative hemorrhage, including surgical damage to blood vessels and hemostatic function disorders (6, 7). However, specific risk factors, and their incidence differ based on the institution as well as the country from which the data are collected (8). The present study aimed to analyze possible risk factors and short-term outcomes for massive postoperative bleeding in patients with ATAAD who underwent aortic repair emergently, we also made a predictive model which could provide details needed for clinical practitioners to avoid or minimize poor outcomes of the procedure.

## Materials and Methods

### Patient Enrollment

This was a single-center, retrospective study, and data were collected from the electronic medical record database of Beijing Anzhen Hospital. A total of 402 consecutive patients who underwent emergent surgery for ATAAD repair during 2019 were enrolled in this study. Patients younger than 18 years old or diagnosed with hereditary connective tissue diseases before were excluded from the study. Each patient was counted only once in the analysis. The Institutional Review Board of Beijing Anzhen Hospital of Capital Medical University approved this retrospective study and waived the need for informed patient consent. The institutional approval number is 202075X.

### Definition of the Massive Bleeding

In this study, the universal definition of perioperative bleeding (UDPB) was used to define massive bleeding (9, 10). The UDPB defines perioperative bleeding by nine clinical events during the surgery or within the first postoperative day. In UDPB, class 3 and 4 bleeding often requires or has already been performed aggressive medical interventions, otherwise, it may lead to extremely serious adverse consequences. Therefore, in this study, class 3 and 4 bleeding was defined as perioperative massive bleeding. Kidney disease: improving global outcomes (KDIGO) grade 3 was used to define the acute kidney injury in this study, and the fraction of inspired oxygen less than 300 was defined as respiratory failure.

### Operative Management

All surgeries were performed *via* median sternotomy under moderate hypothermic circulatory arrest. A dual-stage atriocaval cannula was inserted into the right atrium, and the right axillary artery was isolated routinely for CPB and selected cerebral perfusion. The ascending aorta was clamped at the distal end, and then the patients’ nasopharyngeal temperature was reduced to approximately 25°C. During the cooling, proximal manipulations, such as aortic valve repair, sinus of Valsalva reconstruction, and composite valved graft replacement, would be commenced if necessary. CPB was discontinued when the nasopharyngeal temperature was lower than 25°C, while the selected cerebral perfusion was continued at a rate of approximately 5 to 10 mL × kg^–1^ × min^–1^ toward the right axial artery.

Methods of aortic arc surgery include hemiarch replacement and total arc replacement + frozen elephant trunk (FET); decision-making depends on the general condition of the patient, location of the entry tear, the distal extent of dissection, the occurrence of malperfusion, and intraoperative findings. The hemiarch replacement included arc repair of the distal anastomosis located at the proximal end of the aortic arch or part of the small curvature. In total arc replacement + FET, a stent is inserted as the FET into the descending aorta, and the remaining aortic arch is replaced by the four-branch artificial vessel. More details regarding the surgical procedure have been discussed in our previous studies (11–13).

### Statistical Analysis

Continuous data were presented as means ± standard deviation (SD) with a normal distribution and median (lower quartile, upper quartile) with a non-normal distribution, the categorical variables were described as percentages. For performing comparisons, a Student’s *t*-test or Mann–Whitney *U* test was used for continuous variables, and the Chi-square test or Fisher’s exact test was used for categorical variables. Multivariable regression analyses were conducted to explore relationships between perioperative parameters and severe bleeding using binary logistic modeling techniques. The risk predicting model was built using logistic regression. The area under the receiver operating characteristic curve (AUC) and 95% confidence intervals (CIs) were used to assess a model’s ability to discriminate between high- and low-risk patients. Hosmer–Lemeshow good of fit test was used to evaluate the calibration of the model. *P*-values determined using two-tailed distributions <0.05 were considered statistically significant. All statistical analyses were performed using SPSS 25.0 (IBM SPSS, Armonk, NY, United States) and GraphPad Prism 8.

## Results

### Baseline Characteristics

**FIGURE 1:**
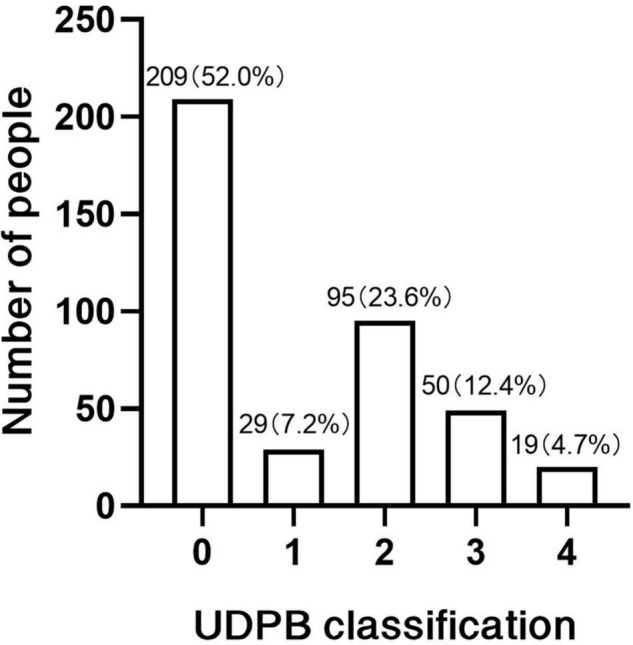
Bleeding classification according to the UDPB.

In this study, 402 patients were enrolled, 69 of them presented with massive postoperative bleeding (17.2%), the detailed classification of massive bleeding was shown in Figure 1. The basic characteristics of patients were summarized in Table 1. The mean age was 52.13 ± 11.96 and 48.73 ± 11.63 years (*P* = 0.029), the mean body mass index (BMI) was 24.99 ± 3.99 in the massive bleeding group and 26.72 ± 7.86 in the non-massive bleeding group (*P* = 0.029), the mean hemoglobin and platelet count in two groups were 129.34 ± 22.26 g, 134.78 ± 18.41 g (*P* = 0.036), 180.72 ± 53.28 109/L, and 198.26 ± 75.57 109/L (*P* = 0.024), separately.

**TABLE 1:** Preoperative characteristics of the study population.

Variables	Massive bleeding group (*n* = 69)	Non-massive bleeding group (*n* = 333)	*P*-value
Male sex	59 (86.8%)	234 (70.3%)	0.005
Age (years)	52.13 ± 11.96	48.73 ± 11.63	0.029
Body mass index	24.99 ± 3.99	26.72 ± 7.86	0.010
Hypertension	57 (82.6%)	277 (83.2%)	0.269
Diabetes mellitus	1 (1.4%)	12 (3.6%)	0.365
Past cerebral infarction	1 (1.4%)	9 (2.7%)	0.553
Preoperative anticoagulant	5 (7.4%)	13 (3.9%)	0.211
Acute cardiac tamponade	1 (1.4%)	6 (1.8%)	0.839
Acute myocardial infarction	1 (1.4%)	6 (1.8%)	0.839
Renal failure	1 (1.4%)	5 (1.5%)	0.974
Malperfusion syndrome	6 (8.7%)	31 (9.3%)	0.872
White blood cell count (×10^9^/L)	12.11 ± 4.36	11.65 ± 4.12	0.404
Red blood cell count (×10^9^/L)	4.38 ± 0.55	4.40 ± 0.74	0.815
Platelet count (×10^9^/L)	180.72 ± 53.28	198.26 ± 75.57	0.024
Mean platelet volume (fL)	10.56 ± 0.90	10.51 ± 1.14	0.053
Hemoglobin (g/L)	129.34 ± 22.26	134.78 ± 18.41	0.036
Prothrombin time (s)	13.62 ± 4.91	12.91 ± 3.16	0.713
International normalized ratio	1.13 (1.04, 1.17)	1.11 (1.04, 1.77)	0.673
D-dimer (ng/mL)	3036.5 (1630, 11970.5)	2040 (790.5, 3229.5)	0.007
Fibrin degradation products (μg/mL)	46.76 (14.68, 95.11)	18.76 (7.33, 37.56)	0.001
Aspartate transaminase level (U/L)	22 (17, 29.5)	23 (17, 35)	0.355
Alanine transaminase level (U/L)	20 (14, 28.5)	22 (15, 38)	0.067
γ-GT (U/L)	24 (14, 35.5)	31 (19, 65.75)	0.058
Serum creatinine (μmol/L)	88.2 (69.72, 129)	84.3 (68.32, 106.22)	0.251
Blood urea nitrogen (mg/dL)	7.83 ± 3.25	7.19 ± 4.39	0.268
Serum lipase (U/L)	12.1 (7.75, 24.1)	12.6 (8.2, 20.65)	0.853
Serum amylase (U/L)	44.45 (36.92, 65.25)	46.1 (35.3, 59.5)	0.735
CK-MB (ng/mL)	1.65 (1.1, 6.67)	1.3 (0.8, 2.97)	0.01
Cardiac troponin I (ng/mL)	0.01 (0, 0.15)	0.01 (0, 0.06)	0.464
Myoglobin (ng/mL)	40.25 (25.72, 96.32)	29.5 (19.82, 49.4)	0.004

*Values are expressed as number (%), median (lower quartile, upper quartile), or mean ± SD.*

All the details of operation data were summarized in Table 2. A total of 298 patients underwent emergent aortic repairs within 24 h of onset (74.1%). In the management of the proximal aorta, 172 patients underwent aortic root replacement (Bentall procedure) (42.8%), 230 patients underwent ascending aorta replacement (58.2%). The management of the aortic arch consists of 2 procedures, 315 patients underwent total arch replacement (78.4%), 60 patients underwent hemiarch replacement (14.9%).

**TABLE 2:** Surgical data of study population.

Variables	Massive bleeding group (*n* = 69)	Non-massive bleeding group (*n* = 333)	*P*-value
Emergent surgery	58 (84.1%)	240 (80.8%)	0.044
Aortic root replacement	32 (46.4%)	140 (42.0%)	0.590
Hemiarch replacement	2 (2.9%)	58 (17.4%)	0.002
Total arch replacement	62 (89.9%)	253 (76.0%)	0.012
Concomitant CABG	6 (8.7%)	25 (7.5%)	0.736
Concomitant vascular bypass	4 (5.8%)	18 (5.4%)	0.896
Concomitant aortic valvuloplasty	1 (1.4%)	10 (3.0%)	0.472
Concomitant mitral valve replacement	1 (1.4%)	1 (0.3%)	0.217
Concomitant abdominal aortic replacement	1 (1.4%)	0 (0)	0.028
Cardiopulmonary bypass time (min)	209.60 ± 43.55	187.83 ± 42.09	<0.001
Aortic cross-clamp time (min)	118.09 ± 26.97	108.44 ± 28.25	0.01
Circulatory arrest time (min)	23.59 ± 11.35	22.94 ± 12.25	0.651

*Values are expressed as number (%) or mean ± SD. CABG, coronary artery bypass graft surgery.*

### Risk Factors of the Massive Bleeding

Based on the definition of the UDPB bleeding classification, a univariate analysis of the patient’s perioperative condition revealed that risk factors associated with massive bleeding after the surgery included aged patients (*P* = 0.029), male patients (*P* = 0.050), emergent surgery (*P* = 0.044), preoperative renal dysfunction (*P* = 0.013), low hemoglobin level (*P* = 0.036), low platelet count (*P* = 0.024), elevated D-dimer level (*P* = 0.045), increased fibrin degradation products (*P* = 0.002), prolonged cardiopulmonary bypass time (*P* < 0.001), prolonged aortic cross-clamp time (*P* = 0.01), and total arch replacement (*P* = 0.012). In a multivariate regression analysis, this study set massive bleeding as the study endpoint and obtained independent risk factors associated with it including male sex (OR = 6.493, *P* < 0.001, 95% CI = 2.429–17.360), elder patients (OR = 1.029, *P* = 0.05, 95% CI = 1.000–1.059), low BMI (OR = 0.879, 95% CI: 0.806–0.957, *P* = 0.003), emergent surgery (OR = 3.112, 95% CI: 1.235–7.843, *P* = 0.016), prolonged cardiopulmonary bypass time (OR = 1.012, 95% CI: 1.004–1.021, *P* = 0.002), lower hemoglobin levels (OR = 0.976, 95% CI: 0.959–0.993, *P* = 0.002), increased D-dimer levels (OR = 1.000, *P* = 0.037), increased fibrin degradation products (OR = 1.019, 95% CI: 1.005–1.033, *P* = 0.008), hemiarch replacement (OR = 5.045, 95% CI: 1.098–23.173, *P* = 0.037), total arch replacement (OR = 14.405, 95% CI: 2.046–101.404, *P* = 0.004) (Table 3).

**TABLE 3:** Risk factors of postoperative massive bleeding.

Variables	OR	*P*-value	95% CI
Male sex	6.493	0.001	2.429–17.360
Elder patients	1.029	0.05	1.000–1.059
BMI	0.879	0.003	0.806–0.957
Emergent surgery	3.112	0.016	1.235–7.843
Prolonged cardiopulmonary bypass time	1.012	0.002	1.004–1.021
Hemoglobin levels	0.976	0.002	0.959–0.993
D-dimer levels	1.000	0.037	1.000–1.002
Fibrin degradation products levels	1.019	0.008	1.005–1.033
Hemiarch replacement	5.045	0.037	1.098–23.173
Total arch replacement	14.405	0.004	2.046–101.404

### Perioperative Outcomes

**FIGURE 2:**
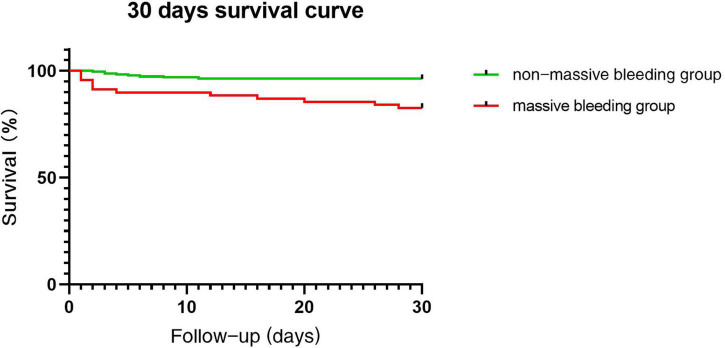
Survival in patients with and without massive bleeding.

In terms of prognosis, 24 patients died in the early stage after surgery, with a mortality rate of 15.9% in the massive bleeding group (11 patients) and 3.9% in the non-massive bleeding group (13 patients, *P* = 0.001), the in-hospital survivorship curve was shown in Figure 2. The average hospitalization time was 17.79 ± 12.73 and 14.54 ± 8.49 days, the average length of stay in the intensive care unit was 4 (2, 8) and 2 (1, 4) days, and the average duration of tracheal intubation was 41 (17.75, 96) and 16 (12, 36) h, respectively. More details were summarized in Table 4.

**TABLE 4:** Short-term outcomes of study population.

Variables	Massive bleeding group (*n* = 69)	Non-massive bleeding group (*n* = 333)	*P*-value
Mortality	11 (15.9%)	13 (3.9%)	0.001
Respiratory failure	28 (41.2%)	70 (21.0%)	0.001
Acute kidney injury	18 (26.5%)	32 (9.6%)	0.001
Endotracheal intubation time (h)	41 (17.75, 96)	16 (12, 36)	0.001
ICU stay (days)	4 (2, 8)	2 (1, 4)	0.001
Cerebrovascular event	7 (10.3%)	30 (9.0%)	0.739
Paraplegia	4 (5.9%)	15 (4.5%)	0.626
Reintubation	9 (13.2%)	31 (9.3%)	0.325
Re-exploration for bleeding	19 (27.5%)	0 (0)	0.001

*Values are expressed as number (%), median (lower quartile, upper quartile), or mean ± SD.*

### The Predicting Model

**FIGURE 3:**
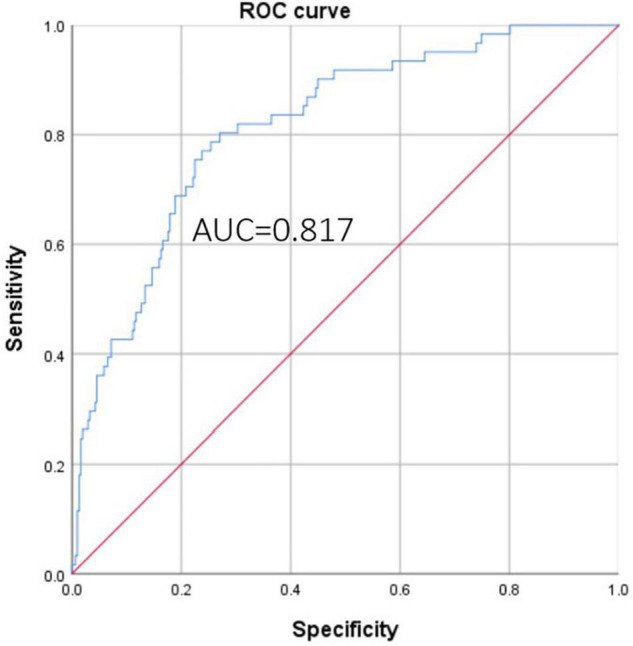
ROC curve of the model for predicting massive bleeding.

**FIGURE 4:**
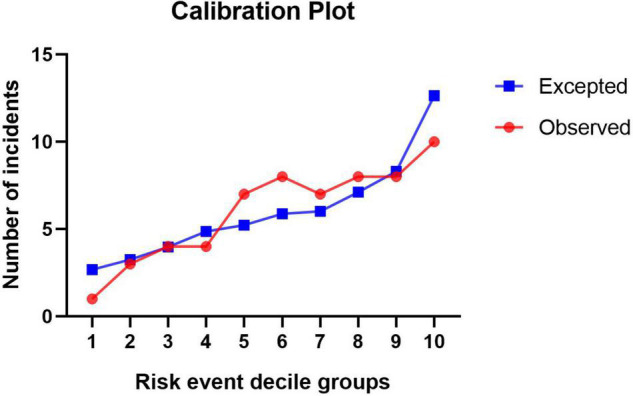
Calibration plot of the model for predicting massive bleeding.

A logistic regression analysis was performed to develop a predicting model in this study, the *R*^2^ was 0.31 and the standardized beta coefficients for the included variables were male sex (1.871), age (0.029), emergent surgery (1.135), hemoglobin level (−0.024), CPB time (0.012), BMI (−0.129), fibrin degradation products (0.019), hemiarch replacement (1.618), and total arch replacement (2.668). The resulting regression equation to predict the mean ascending aorta length was:

Logit(*P*) = −3.226 + sex [*m* = 1, *f* = 0] × 1.871 + age (years) × 0.029 + emergent surgery [*y* = 1, *n* = 0] × 1.135 – hemoglobin level (g/L) × 0.024 + CPB time (min) × 0.012 – BMI × 0.129 + fibrin degradation products (μg/mL) × 0.019 + hemiarch replacement [*y* = 1, *n* = 0] × 1.618 + total arch replacement [*y* = 1, *n* = 0] × 2.668 (14). In terms of evaluating the discrimination of the model, the area under the curve (AUC) was 0.817 (95% CI: 0.760–0.873, *P* < 0.001, Figure 3). In the Hosmer–Lemeshow goodness-of-fit test, the model demonstrated good calibration, the Hosmer–Lemeshow χ^2^ was 5.281, *P* = 0.727 > 0.05, and the calibration plot was shown in Figure 4.

## Discussion

Postoperative bleeding and the need for blood product transfusion are quite common after aortic surgery (3). At the same time, massive bleeding may lead to numerous complications such as re-exploration, which is associated with increased mortality (15). In previous studies, the definition of massive bleeding has varied depending on the countries and centers, and is less often measured by a uniform standard. In this study, the UDPB bleeding classification was used to measure postoperative bleeding in patients with ATAAD, with class 3–4 bleeding requiring aggressive clinical intervention and therefore defined as massive bleeding by this study. The proportion of bleeding at all levels in our center is generally consistent with previous studies.

Gender was considered an independent risk factor for massive postoperative bleeding in patients with aortic type A coarctation, along with low body mass index. In general, low weight patients are associated with female patients, but the present study concluded the opposite, that male patients are more likely to have massive postoperative bleeding after aortic repair surgery, which is consistent with the results of many previous trials (16–18). The possible reason for this is that in our study, most male patients were in middle age, with poorly controlled hypertension, which may lead to difficulty in intraoperative hemostasis, another reason might be the rate of fibrin production as well as the intensity of thrombosis is higher in healthy adult females than in healthy males (19). At the same time, elderly patients have a higher risk of massive postoperative bleeding compared to younger patients, probably because the vascular condition is worse in elderly patients, which makes the operation more difficult. The coagulation mechanism of elderly patients is not as well developed as that of younger patients, which makes them more prone to massive postoperative bleeding, and therefore the prognosis of elderly patients needs to be considered more comprehensively before and during the surgery.

In this study, elevated D-dimer and preoperative low hemoglobin status were both strongly associated with postoperative bleeding, suggesting that preoperative assessment of coagulation mechanisms is particularly important, and that D-dimer, as a degradation product of fibrinolysis of blood clots, is often indicative of fibrin production and lysis, commonly in response to inflammation, trauma, tumor, recent surgery, and acute aortic syndrome or pulmonary embolism (20, 21). Previous studies have shown that elevated D-dimer is significantly and positively associated with the severity of the tear in aortic dissection, and it has therefore been mentioned in several studies that patients with significantly elevated D-dimer tend to undergo more complex procedures, but this is accompanied by difficulties in intraoperative hemostasis and heavy bleeding in the early postoperative period, which in turn affects the early prognosis of the patient (22, 23). Itagaki et al. found that an increase in D-dimer was accompanied by a decrease in platelet count, further demonstrating the value of D-dimer as a predictor of bleeding risk in the early stages of the disease (24). Our study did not find a significant effect of preoperative anticoagulant use on postoperative massive bleeding, probably due to the young age of the patients with type A aortic dissection seen at our center and the very limited number of patients with coronary artery disease on antiplatelet agents. Most of the patients on anticoagulants were misdiagnosed at other hospitals and underwent coronary angiography or were treated as myocardial infarctions; the number of such patients was so small that it did not significantly affect the statistical results.

Although it was shown in this study that emergent surgery raises the risk of massive postoperative bleeding, and similar results were obtained in other studies such as Zindovic et al. Emergent surgical treatment to save the patient’s life is still the first choice compared to the risk of death from a ruptured aortic coarctation aneurysm that the patient is waiting to face (25). With regard to the choice of procedure, a single-center retrospective study by Xue et al. covering 958 patients suggested that differences in arch procedure alone did not result in significant mortality differences and that it was the preoperative status that really affected the prognosis of patients with type A dissection, which is generally consistent with the results of the present study (26). Our study suggests that either hemiarch replacement or total arch replacement increases the likelihood of massive postoperative bleeding compared to ascending aortic surgery alone, but the surgical approach still needs to be tailored to the patient.

The results of this study showed a significant difference in the short-term prognosis of patients undergoing aortic repair with the presence or absence of massive bleeding, with patients who had massive bleeding in the study having a worse prognosis than those in the non-massive bleeding group. Our results showed that there was a statistical difference in mortality between the two groups, and the main causes of death in the massive bleeding group included cerebrovascular accident and postoperative multiorgan failure, of which multiorgan failure was more closely related to massive bleeding, blood volume deficiency, and ischemia-reperfusion injury.

This study found that among patients who presented with massive postoperative bleeding, there was a significant increase in the need for continuous renal replacement therapy due to impaired renal function. Inadequate preoperative as well as intraoperative renal perfusion, activation of the inflammatory response triggered by postoperative bleeding, postoperative low cardiac output and hemodynamic changes are all possible predisposing factors for the occurrence of postoperative acute kidney injury (27, 28).

In terms of the respiratory system, this study suggests that the duration of tracheal intubation was significantly longer in patients who presented with massive postoperative bleeding and required longer periods of ventilator assistance. The possible causes include prolonged CPB time, endotoxemia due to poor visceral perfusion, ischemia-reperfusion injury to the lungs, surgical trauma, or the use of protamine, an inflammatory response that can cause damage to the lungs, and inappropriate postoperative ventilatory parameters and fluid management may exacerbate this damage (29). The amount of blood product infusion associated with massive bleeding can also be significantly elevated, and some studies have shown that blood product infusion significantly increases the incidence of respiratory distress after cardiac surgery. Moreover, the low blood pressure caused by massive bleeding can also make postoperative fluid management very difficult, and excessive fluid rehydration may cause pulmonary edema further aggravating the blow to the lungs (30).

Several previous studies have discussed predicting the risk of bleeding after cardiac surgery, but most of these studies were limited to coronary surgery (14, 31, 32). In this study, we used a logistic regression model to predict the risk of massive bleeding after aortic repair in ATAAD patients, the model was performed well and showed good discrimination (AUC = 0.817) and calibration. Since multiple adverse prognoses are associated with massive bleeding, patients at high risk should be more carefully assessed preoperatively and treated with a reasonable treatment plan.

The limitation of this study was the retrospective nature of the analysis. Despite the use of multivariable analyses to limit the effects of confounding variables, the effects of baseline differences as confounders could not be ruled out. Postoperative bleeding may also be related to many surgical factors such as different surgical strategies, insufficient anastomosis, improper surgical manipulation, and suture lines. Further studies investigating mechanisms underlying these risk factors for massive bleeding are required. Risk factors for each set of criteria were not completely the same, and more clinical trials are needed to identify which criteria are more favorable in clinical use. At the same time, although the predicting model showed good discrimination and calibration in our study, more data from multiple centers may validate the model in further studies.

## Conclusion

The present study reported several risk factors for massive bleeding in patients with ATAAD who underwent emergent aortic repair, including gender, emergent surgery, increased D-dimer level, increased CPB time, and use of a complex surgical strategy. Massive bleeding may lead to higher mortality, longer stay in the intensive unit, and longer hospitalization. Our predicting model showed good discrimination and calibration, for patients who were evaluated as having a high risk of massive bleeding, surgeons should choose a suitable surgical strategy to prevent bleeding complications.

## Data Availability Statement

The original contributions presented in this study are included in the article/supplementary material, further inquiries can be directed to the corresponding author.

## Ethics Statement

The Institutional Review Board of Beijing Anzhen Hospital of Capital Medical University approved this retrospective study and waived the need for informed patient consent. The institutional approval number is 202075X.

## Author Contributions

C-HZ was responsible for the conceptualization, data collection, statistical analysis, and writing the draft. Y-LZ and Y-PG were responsible for the statistical analysis. J-MZ was responsible for the conceptualization, methodology, and investigation. H-OH, Z-YQ, C-NL, and Y-PG were responsible for the investigation. All authors contributed to the article and approved the submitted version.

## Conflict of Interest

The authors declare that the research was conducted in the absence of any commercial or financial relationships that could be construed as a potential conflict of interest.

## Publisher’s Note

All claims expressed in this article are solely those of the authors and do not necessarily represent those of their affiliated organizations, or those of the publisher, the editors and the reviewers. Any product that may be evaluated in this article, or claim that may be made by its manufacturer, is not guaranteed or endorsed by the publisher.
